# Notes and News

**Published:** 1891-08-29

**Authors:** 


### Notes and News.

Collected and Communicated.

The Irish Distressed Ladies' Fund has been at work some time now, and from the report of the society for the past year we gather that some of the interest shown in the work at its outset has fallen off somewhat. Not so the need of those for whose aid it was commenced. In fact, the condition of many of the helpless sufferers from the rentless state of many Irish estates is as bad, if not worse, than it was some years ago. Is is difficult to convey a picture of the abject want which many poor Irish ladies are experiencing. The Fund has done much, but some of its operations are being curtailed on account of a diminution of subscriptions. So much really beneficial relief has been distributed by the Society that it seems a pity any of its usefulness should be curtailed. Besides gratuitous help, the Society assists by procuring work for those ladies skilled in needlework, and this part of the work is in a flourishing condition. There is a depôt for the sale of articles of all sorts at 17, North Audley Stret, and where orders for all kinds of needlework can be received. For those who object to give in money, here is an opportunity of forwarding a most useful charity and procuring a really excellent return.

The Congress of Hygiene has brought before our notice a society that has been doing most useful and steady work without any great flourish of trumpets or desire to hold up its good deeds to public view. We allude to the Ladies' Sanitary Association, the patroness of which is the Princess of Wales, an ever ready supporter of all good works. The society was established as far back as 1856, which shows that the necessity of sanitation was earlier realised than we are inclined to think. Dr. Roth in the year mentioned pointed out that womanly influence would be a powerful agent in effecting improvements in sanitary matters. His idea was taken up with eagerness by a few ladies, who formed the nucleus of the present society. These ladies combined to extend and popularise sanitary knowledge, and help was given them by medical men of the first rank. They started by publishing short, simple explanations of health principles, in the guise of narratives. During the first year 32,500 copies of these tracts were distributed gratuitously. Since then nearly two million tracts have been circulated. The society has developed its lines of teaching year by year, and lectures, classes, and cottage meetings were organised as time went on. Nor has the influence of recreation been overlooked. Gymnastics were placed within the reach of many poor children, whilst others were taken in parties several times a week into the parks, open spaces, and fields. And last, but not least, a depôt was established, where cleansing utensils, such as baths, pails, &c., were lent, and soap, disinfectants, &c., were given away. The society is always adding to its sphere of usefulness, and a constant effort to remedy existing evils and establish healthy modes of living amongst the poorer classes is made.

It is of not unfrequent occurrence that when one member of a family is ordered to winter abroad the great difficulty arises of who is to accompany them. They cannot go alone to hotel or pension, and no friend or member of the family is able to accompany them. In such cases establishments on not too large a scale, where home comforts and nursing, if necessary, are procurable, are great blessings in foreign health resorts. We are, therefore, glad to hear that Mrs. Dan Norris is likely to take a villa in October next, probably at San Remo, Riviera, where she would receive patients who are ordered to winter abroad. Mrs. Norris (*née* Williams) was one of the nurses' staff during the Egyptian campaign, and late Matron of St. Mary's Hospital, Paddington. She is quite an authority in the nursing world, and is authoress of the series of manuals known as "Norris's Nursing Notes." Mrs. D. Norris's London address is 2, Dennington Park Mansions, West Hampstead, where early application should be made to her by letter.

A Dublin paper gives us a short account of the history and successes of St. Vincent's Hospital as a medical teaching institution. The results shown in a prospectus now issued are sufficiently striking to justify the claim of the hospital to be considered as regards the attendance of students foremost among the teaching institutions of Dublin. The average annual attendance of students for the past six years has been 104—a record without parallel. The hospital never was so fully equipped as now for both its charitable and teaching work, and the city may fairly boast of it as a noble structure, admirably managed, and officered in every department by the most experienced surgeons and physicians. The Hospital contains 160 beds, constantly occupied. During the nine months ending June 30th, 1891, 581 medical and 455 surgical cases, together with 110 cases of diseases of the eye and ear, have been treated within the wards. The whole practice of the hospital is available for clinical instruction. The list of past members of the staff is a very large and most distinguished one, and sufficient in itself to verify and put emphasis upon every statement in the prospectus.

The annual report of the Army Medical Department, which has just been published, shows that, taking the statistics of the three divisions of the kingdom separately, and comparing the ratios with the corresponding results in the previous year, it is found that in England there was a decrease of 20 8 per 1,000 in the ratio of admissions into the hospitals, one of 1·11 in the ratio of mortality, and one of 4·13 in that of constantly sick. Compared with the average ratios for the preceding three years the decrease in the admission rate is equal to 63·8, that in the death rate to ·95, and that in the constantly sick rate to 4·48 per 1,000. The average sick time to each soldier is shorter by 1·63 days, and the average duration of each case of sickness by ·35 a day. In Scotland the admission ratio was increased by 15 per 1,000 as compared with that in the previous year, but the death rate shows a decline of 1·66, and the rate of constant inefficiency through sickness a decline of 2·22. In Ireland the admission ratio shows an increase of over 14·0 per 1,000 as compared with the corresponding ratio in the previous year, the constantly sick rate is fractionally higher, but there is a decrease of ·75 in the mortality rate. For the whole kingdom the admission ratio for the year shows a decline of 10·5 per 1,000 as compared with the corresponding rate of the previous year; in the same comparison the death rate shows a decrease of ·95, and the ratio of constant inefficiency through sickness a decrease amounting to 2·97 per 1,000. The total loss by death and final discharge from the service on account of medical unfitness amounted to 2,089 men, which is equal to a ratio of 20·46 per 1,000 of strength, being lower than the corresponding ratio in the preceding year by ·97 per 1,000.

